# A Glycosylphosphatidylinositol-Anchored Carbonic Anhydrase-Related Protein of *Toxoplasma gondii* Is Important for Rhoptry Biogenesis and Virulence

**DOI:** 10.1128/mSphere.00027-17

**Published:** 2017-05-17

**Authors:** Nathan M. Chasen, Beejan Asady, Leandro Lemgruber, Rossiane C. Vommaro, Jessica C. Kissinger, Isabelle Coppens, Silvia N. J. Moreno

**Affiliations:** a Center for Tropical and Emerging Global Diseases, University of Georgia, Athens, Georgia, USA; b Department of Infectious Diseases, University of Georgia, Athens, Georgia, USA; c Instituto Nacional de Metrologia, Inmetro-RJ, UFRJ, Rio de Janeiro, Brazil; d Instituto de Biofísica Carlos Chagas Filho, UFRJ, Rio de Janeiro, Brazil; e Department of Genetics, University of Georgia, Athens, Georgia, USA; f Institute of Bioinformatics, University of Georgia, Athens, Georgia, USA; g Department of Molecular Microbiology and Immunology, Johns Hopkins Bloomberg School of Public Health, Baltimore, Maryland, USA; h Department of Cellular Biology, University of Georgia, Athens, Georgia, USA; Indiana University School of Medicine

**Keywords:** carbonic anhydrase, infectivity, *Toxoplasma gondii*, glycosylphosphatidylinositols, organelle structure, rhoptry

## Abstract

Carbonic anhydrase-related proteins (CARPs) have previously been described as catalytically inactive proteins closely related to α-carbonic anhydrases (α-CAs). These CARPs are found in animals (both vertebrates and invertebrates) and viruses as either independent proteins or domains of other proteins. We report here the identification of a new CARP (TgCA_RP) in the unicellular organism *Toxoplasma gondii* that is related to the recently described η-class CA found in *Plasmodium falciparum*. TgCA_RP is posttranslationally modified at its C terminus with a glycosylphosphatidylinositol anchor that is important for its localization in intracellular tachyzoites. The protein localizes throughout the rhoptry bulbs of mature tachyzoites and to the outer membrane of nascent rhoptries in dividing tachyzoites, as demonstrated by immunofluorescence and immunoelectron microscopy using specific antibodies. *T. gondii* mutant tachyzoites lacking *TgCA_RP* display a growth and invasion phenotype *in vitro* and have atypical rhoptry morphology. The mutants also exhibit reduced virulence in a mouse model. Our results show that TgCA_RP plays an important role in the biogenesis of rhoptries.

**IMPORTANCE** *Toxoplasma gondii* is an intracellular pathogen that infects humans and animals. The pathogenesis of *T. gondii* is linked to its lytic cycle, which starts when tachyzoites invade host cells and secrete proteins from specialized organelles. Once inside the host cell, the parasite creates a parasitophorous vacuole (PV) where it divides. Rhoptries are specialized secretory organelles that contain proteins, many of which are secreted during invasion. These proteins have important roles not only during the initial interaction between parasite and host but also in the formation of the PV and in the modification of the host cell. We report here the identification of a new *T. gondii* carbonic anhydrase-related protein (TgCA_RP), which localizes to rhoptries of mature tachyzoites. TgCA_RP is important for the morphology of rhoptries and for invasion and growth of parasites. TgCA_RP is also critical for parasite virulence. We propose that TgCA_RP plays a role in the biogenesis of rhoptries.

## Abstract (precis)

*Toxoplasma gondii* is an intracellular pathogen that infects humans and animals. The pathogenesis of *T. gondii* is linked to its lytic cycle, which starts when tachyzoites invade host cells and secrete proteins from specialized organelles. Once inside the host cell, the parasite creates a parasitophorous vacuole (PV) where it divides. Rhoptries are specialized secretory organelles that contain proteins, many of which are secreted during invasion. These proteins have important roles not only during the initial interaction between parasite and host but also in the formation of the PV and in the modification of the host cell. We report here the identification of a new *T. gondii* carbonic anhydrase-related protein (TgCA_RP), which localizes to rhoptries of mature tachyzoites. TgCA_RP is important for the morphology of rhoptries and for invasion and growth of parasites. TgCA_RP is also critical for parasite virulence. We propose that TgCA_RP plays a role in the biogenesis of rhoptries.

## INTRODUCTION

The parasite* Toxoplasma gondii* is an important cause of congenital disease and infection in immunocompromised patients. The parasite can cause ocular uveitis in immunocompetent individuals (1), pneumonia or encephalitis in immunodeficient patients (2), and serious malformations in congenitally infected children (3). *T. gondii* utilizes a complex secretory system, which is essential for host cell invasion and the establishment of a parasitophorous vacuole (PV). This system is composed of multiple organelles: rhoptries, micronemes, and dense granules. These organelles release their contents when the parasite invades a host cell. Rhoptries are club-shaped organelles which share some characteristics with secretory lysosomes (4) and are comprised of two distinct regions termed the rhoptry neck and the rhoptry bulb, each compartment containing distinct protein constituents (5, 6).

The α-carbonic anhydrases (α-CAs) are zinc metalloenzymes found in a variety of organisms that catalyze the reversible hydration of CO_2_, which is important for a number of biological functions, including respiration, photosynthesis, renal tubular acidification, and bone resorption (7–9). In addition to these enzymatically active CAs, there are CA isoforms known as carbonic anhydrase-related proteins (CARPs), which share sequence and structural similarity to active CAs but lack enzymatic activity. CARPs can exist either as independent proteins or as domains of other proteins. Their lack of activity is due to the absence of one or more histidine amino acids that are required in the active site for zinc ion (Zn^2+^) coordination (9). CARPs have been described in vertebrates, insects, nematodes, and viruses (9), but there are no reports of their presence in early branches of the tree of life.

The *Plasmodium falciparum* carbonic anhydrase (PfCA; PF3D7_1140000, Genbank accession no. CZT99063.1) was recently characterized and classified into a new class of CAs, η-CAs. An interesting peculiarity of the PfCA sequence is the presence of the amino acid glutamine in the Zn^2+^ coordination site, which replaces one of the three canonical histidine residues. PfCA is catalytically active in spite of this substitution (10, 11).

In the present work, we investigated one of the annotated carbonic anhydrase proteins of *T. gondii* (TGME49_297070). Analysis of the sequence of the *Toxoplasma* protein showed that it is more similar to PfCA than to the α-CAs. We characterize and localize TgCA_RP in *T. gondii* tachyzoites and analyze the phenotypic profile of mutant parasites (*Δcarp*) isolated after the clean deletion of the *TgCA_RP* gene.

## RESULTS

### Identification of a carbonic anhydrase-related protein, TgCA_RP.

A gene originally annotated as a carbonic anhydrase and subsequently as a hypothetical protein (TGME49_297070, NCBI accession no. XP_002371137.1) was cloned and sequenced. The open reading frame (ORF) of the annotated gene encodes a protein of 519 amino acids (aa) with a predicted molecular mass of 58 kDa and an isoelectric point of 6.29. We named the gene product *T. gondii* carbonic anhydrase-related protein (TgCA_RP), because close analysis of the amino acid sequence showed that two histidine (His) residues typically required for activity (see below) were replaced by a phenylalanine and a glutamine. In addition, recombinant TgCA_RP was inactive (see below). Analysis of the TgCA_RP amino acid sequence revealed that it contained an α-carbonic anhydrase domain (amino acids 121 to 445) and a predicted signal peptide (amino acids 1 to 39) at its N terminus.

**FIG 1:**
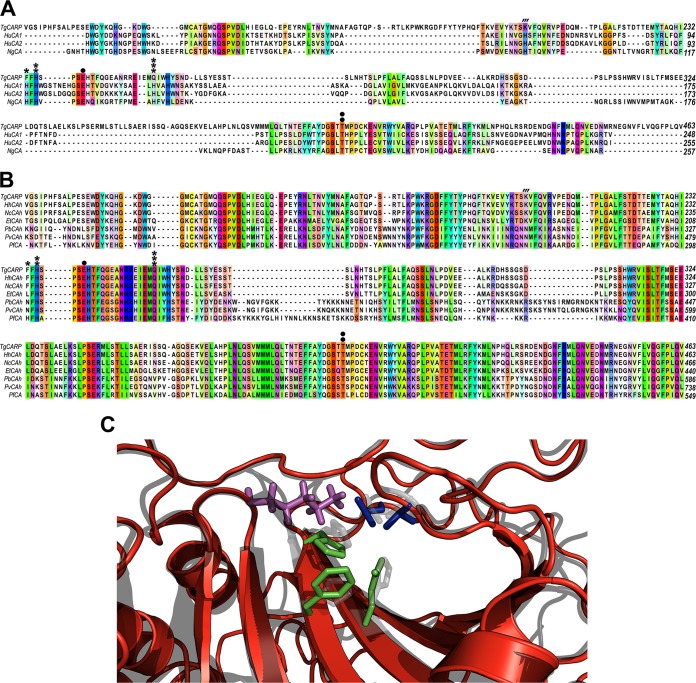
TgCA_RP is most similar to the η-class carbonic anhydrase PfCA. (A) Sequence alignment of the amino acid sequence of TgCA_RP with several α-CAs (human carbonic anhydrase 1 [HuCA1], human carbonic anhydrase 2 [HuCA2], and *Neisseria gonorrhoeae* carbonic anhydrase [NgCA]) highlighting several distinct features. The canonical α-CA Zn^2+^ coordination site, which consists of three histidine (H) residues, is indicated with the symbols *, **, and ***. TgCA_RP shows two substitutions in this domain: a phenylalanine (F) replaces the first histidine (*) and a glutamine (Q) replaces the third conserved histidine (***). The two gateway amino acids, indicated with the symbols • and ••, glutamic acid and threonine, are conserved. Histidine^64^ (′′′), proposed to act as a proton shuttle in HuCA1, HuCa2, and NgCA, is replaced in TgCA_RP by a Lys (K). (B) Multiple sequence alignment of the TgCA_RP amino acid sequence with coccidian (*Neospora caninum* [NcCAh] and *Eimeria tenella* [EtCAh]) and piroplasm (*Plasmodium berghei* [PbCAh], *Plasmodium yoelii* [PyCAh], and *Plasmodium falciparum* [PfCA]) orthologs shows shared features. The proton shuttle histidine indicated with **′′′** is replaced by lysine (K) in the coccidian sequences (TgCA_RP, NcCA, and EtCA) and by asparagine (N) in the Aconoidasida sequences (PyCA, PbCA, and PfCA). The three histidine residues of the Zn^2+^ coordination domain are indicated with the symbols *, **, and ***. The first histidine is conserved in PfCA, but it is replaced by a phenylalanine (*TgCA_RP1* and *NcCAh*), leucine (L) (*EtCAh*), or asparagine (*PbCAh* and *PyCAh*) in apicomplexan orthologs. The gatekeeper residues are indicated with the symbols • and ••, and both the glutamine and threonine are conserved among all members of this group. The orange lane indicates the GPI attachment consensus sequence. (C) Predicted structural alignment of the predicted TgCA_RP α-CA domain (red) with human carbonic anhydrase 2 (gray) showing the important active site residues referenced above in TgCA_RP, corresponding to the α-CA proton shuttle (Lys^201^, purple), gateway residues (Glu^239^ and Thr^414^, blue), and Zn^2+^ coordination residues (Phe^233^, His^235^, and Gln^254^, green).

Alignment of the amino acid sequence of TgCA_RP with those of other α-CA sequences (Fig. 1A; see also Table S1 in the supplemental material) and with orthologous sequences from other apicomplexan parasites (Fig. 1B) illustrates that TgCA_RP is more similar to the *Plasmodium* η-CA, PfCA (PF3D7_1140000), than the α-CAs (Fig. 1). The position of TgCA_RP in a neighbor-joining tree of alpha, beta, eta, and zeta family CAs supports this conclusion (Fig. S2). TgCA_RP also shares several important features with PfCA (12). The canonical α-CA zinc coordination domain consists of three histidines identified by their position in the human carbonic anhydrase 2 (HuCA2) sequence (His^94^, His^96^, and His^119^) (Fig. 1A, *, **, and ***). The third histidine (His^119^) of the zinc coordination domain is replaced with a glutamine (Gln) in the η**-**class enzyme PfCA (Gln^320^, Fig. 1B, ***) (11). In the case of TgCA_RP, two of the three histidines of the Zn^2+^ coordination domain are substituted. Like PfCA, the position of the third histidine is occupied by a Gln (Gln^254^, Fig. 1A, ***), but in addition, the first His is changed to a phenylalanine (Phe^233^, Fig, 1A, *). These two substitutions in an essential domain for Zn^2+^ binding indicated that TgCA_RP would be inactive (13) (Fig. 1A and B). We compared the Zn^2+^ coordination domain of TgCA_RP with the same domain of other apicomplexan CA orthologs (Fig. S1). *Neospora caninum* (NcCAh), *Eimeria tenella* (EtCAh), *Theileria parva* (TpCAh), and *Theileria equi* (TeCAh) show a replacement of the first His in the Zn^2+^ binding domain with Phe, Leu, or Asp (Phe^236^, Leu^209^, Asp^200^, and Asp^196^, respectively). The *Plasmodium berghei* (PbCAh) and *Plasmodium yoelii* (PyCAh) sequences replaced the His in the Zn^2+^ coordination domain with asparagine (Asn^328^ and Asn^480^, Fig. 1A, *). PfCA is, the only protein of this group that possesses a histidine in this position. These substitutions, specifically with asparagines, do not necessarily preclude activity. Previously, Lesburg et al. (14) substituted an asparagine for His^94^ in HuCA2, and the enzyme retained activity, although activity was markedly reduced (14). It is also possible that PfCA is the only enzymatically active carbonic anhydrase of this group and this question will be answered only once the other enzymes are characterized. TgCA_RP also shows other features that define the η class. The gateway residues Glu^106^ and Thr^199^ in HuCA2 are responsible for the orientation of the substrate during catalysis. Both TgCA_RP (Glu^239^ and Thr^414^) and PfCA (Glu^305^ and Thr^500^) are predicted to have these gateway residues in the appropriate position for substrate orientation (14, 15) (Fig. 1C, blue amino acids). TgCA_RP and PfCA lack the His^64^ proton shuttle present in most α-CA active sites, which is responsible for increased catalysis via the transport of protons from catalytic intermediates to the environment (Fig. 1A and B, ′′′) (16–18). This was also the case for the other apicomplexan orthologs that were aligned (Fig. 1B). An alignment of the I‑TASSER predicted structure of the putative TgCA_RP CA domain (amino acids 121 to 446) (19–21) with the crystal structure of HuCA2 (22) illustrates the above features and their location in the predicted active site (Fig. 1C).

10.1128/mSphere.00027-17.1 FIG S1  Sequence alignment of the predicted CA Zn^2+^ coordinating region of *TgCA_RP* with other apicomplexan orthologs (Table S1). The canonical CA Zn^2+^ coordination site consists of three histidines (H), which are indicated with the symbols *, **, and ***. The first position (*) has the most varied amino acid composition. PfCA is the only one that possesses a His in this position. All of the orthologs have a His in the second position (**) and a glutamine in the third position (***). Download FIG S1, PDF file, 0.2 MB. Copyright © 2017 Chasen et al. 2017 Chasen et al. This content is distributed under the terms of the Creative Commons Attribution 4.0 International license.

10.1128/mSphere.00027-17.2 FIG S2  Unrooted tree showing that TgCARP is more closely related to PfCA1 and other putative η-CAs than the α-CAs. A T-Coffee alignment, trimmed by the TrimAI automated1 algorithm, was used, and the tree was generated using the program Geneious, with the neighbor-joining Jukes-Cantor algorithm. The tree was then bootstrapped. Branches are labeled with their bootstrap values. Branch length is representative of evolutionary distance. Download FIG S2, PDF file, 0.1 MB. Copyright © 2017 Chasen et al. 2017 Chasen et al. This content is distributed under the terms of the Creative Commons Attribution 4.0 International license.

10.1128/mSphere.00027-17.6 TABLE S1  Amino acid sequences used for the alignment with TgCA_RP and tree generation. Download TABLE S1, PDF file, 0.2 MB. Copyright © 2017 Chasen et al. 2017 Chasen et al. This content is distributed under the terms of the Creative Commons Attribution 4.0 International license.

Two prediction systems for glycosylphosphatidylinositol (GPI)-anchored proteins, PredGPI (23) and GPI‑SOM (24), predicted that TgCA_RP has a C-terminal GPI anchor cleavage/attachment consensus sequence (Table S2). We also analyzed the sequences of TgCA_RP orthologs from related apicomplexan parasites. All of the orthologs, except for PfCA of *P. falciparum*, were predicted to contain GPI anchor attachment sites, including orthologs of *P. yoelii* and *P. berghei* (Table S2) (23, 24). These predictions suggest that the GPI anchor modification of TgCA_RP and its orthologs may be conserved from a common ancestor of the Apicomplexa.

10.1128/mSphere.00027-17.7 TABLE S2  Sequences of TgCA_RP and orthologs from other apicomplexans were analyzed for their likelihood of being GPI anchored, by using the prediction servers Pred-GPI and GPI-SOM. All orthologs were predicted to likely be GPI anchored, except for the ortholog in *Plasmodium falciparum* (PfCA). Download TABLE S2, PDF file, 0.03 MB. Copyright © 2017 Chasen et al. 2017 Chasen et al. This content is distributed under the terms of the Creative Commons Attribution 4.0 International license.

To investigate the CA activity of TgCA_RP, we expressed two truncated forms, rTgCA_RPa (amino acids [aa] 121 to 445) and rTgCA_RPb (aa 94 to 488), in *Escherichia coli*. The truncated proteins were soluble, unlike the full-length protein, which was expressed and purified for antibody generation (see below). rTgCA_RPa contains the predicted CA domain, and rTgCA_RPb includes, in addition, the N-terminal region downstream of a cleavage motif, SXL↓Q (^91^SLLQ^94^), previously characterized in toxolysin, a rhoptry metalloprotease (25). Both fragments were cloned in the Pet32Lic/EK vector, and the resulting constructs were transformed into *E. coli* for expression. Nickel affinity purification of soluble protein products followed with the aim of measuring rTgCA_RP catalytic activity. Two protocols for activity were used: hydrolysis of *p*-nitrophenyl acetate (pNPA) (26) and in-gel CO_2_ hydration assays (27). Commercially available bovine carbonic anhydrase (Sigma catalog no. C3934) was used as a positive control for activity. We were unable to detect any measurable activity from rTgCA_RPa or rTgCA_RPb when measured at various pHs (pH 6.0 to 8.0) and buffer compositions (Tris-HCl, Tris-SO_4_, Tricine-KOH, HEPES-KOH, morpholineethanesulfonic acid [MES], and morpholinepropanesulfonic acid [MOPS]) (data not shown). It was reported that site-directed mutagenesis of the Zn^2+^ coordination site restored enzymatic activity of some α-type CARPs (28, 29). We performed site-directed mutagenesis of rTgCA_RPa and rTgCA_RPb to alter the predicted Zn^2+^ coordination residues to the His^94^-His^96^-His^119^ of the α-CAs (as in HuCA2) or to His^299^-His^301^-Glu^320^ as in PfCA, but these alterations did not restore activity to rTgCA_RP as measured with either activity assay (data not shown). In summary, sequence analysis and experimental results indicated that TgCA_RP lacks activity. However, we were unable to restore its activity by mutating specific amino acids of rTgCA_RP, making it difficult to definitively state that TgCA_RP is inactive.

### TgCA_RP localized to the rhoptry bulbs.

**FIG 2:**
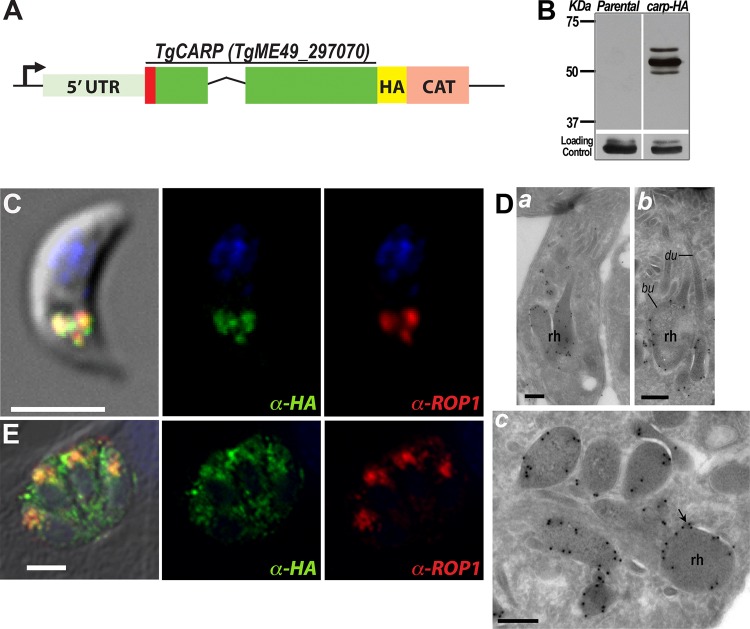
C-terminal tagging of TgCA_RP and its localization to the rhoptry bulbs. (A) Scheme showing the modified *TgCA_RP* locus, depicting the endogenous promoter (arrow), 5′ UTR, coding regions, predicted signal peptide (red), exons (green), C-terminal HA tag (yellow), and selection marker (pink; CAT). (B) Western blot analysis of lysates obtained from tachyzoites of the *Δku80* (parental) and *TgCA_RP-HA* clonal cell lines shows bands of approximately the predicted molecular mass (58 kDa). (C) Immunofluorescence assay (IFA) of TgCA_RP-HA in an extracellular tachyzoite showing colocalization of anti-HA and anti-ROP1, a rhoptry bulb marker. (D) Cryo-immunoelectron microscopy of an extracellular tachyzoite using anti-HA shows localization of TgCA_RP-HA to rhoptry bulbs (bu) as evidenced on parasite longitudinal sections (a and b) and association with the membrane (arrow, c). rh, rhoptry; du, duct. (E) IFA of intracellular tachyzoites showing partial colocalization between anti-HA and anti-ROP1. Punctate and dispersed labeling of anti-HA throughout the parasite cytosol and parasitophorous vacuole (PV) is observed. Bars, 3 µm (C and E) and 150 nM (D).

To investigate the localization of TgCA_RP, an *in situ* epitope-tagging technique was used to avoid the problem of overexpression and potential abnormal distribution of the tagged protein (Fig. 2A). Western blot analysis of a clonal parasite line expressing *TgCA_RP-HA* showed three bands around the predicted molecular mass (58 kDa) of TgCA_RP (Fig. 2B). Immunofluorescence analysis experiments with the parasite clone expressing *TgCA_RP-HA* showed labeling of rhoptry bulbs in extracellular tachyzoites (Fig. 2C, green), determined by colocalization with the rhoptry bulb marker anti-ROP1 (Fig. 2C, red). Localization to rhoptries of TgCA_RP was previously done by expressing an extra copy of the gene with a c-Myc tag (30). To provide fine details of TgCA_RP localization, we performed cryo-immunoelectron microscopy of *TgCA_RP-HA* extracellular tachyzoites. Gold particles were observed in rhoptries (Fig. 2D, a), mainly detected at the basal bulbous portion of these organelles (Fig. 2D, b) and at the limiting membrane (Fig. 2D, c). In intracellular tachyzoites, TgCA_RP-HA showed more diffuse and less specific localization, and antihemagglutinin (anti-HA) labeled other uncharacterized structures, in addition to the rhoptries (Fig. 2E). Interestingly, the morphology of the rhoptries appeared to be affected by the expression of TgCA_RP-HA (Fig. 2E).

**FIG 3:**
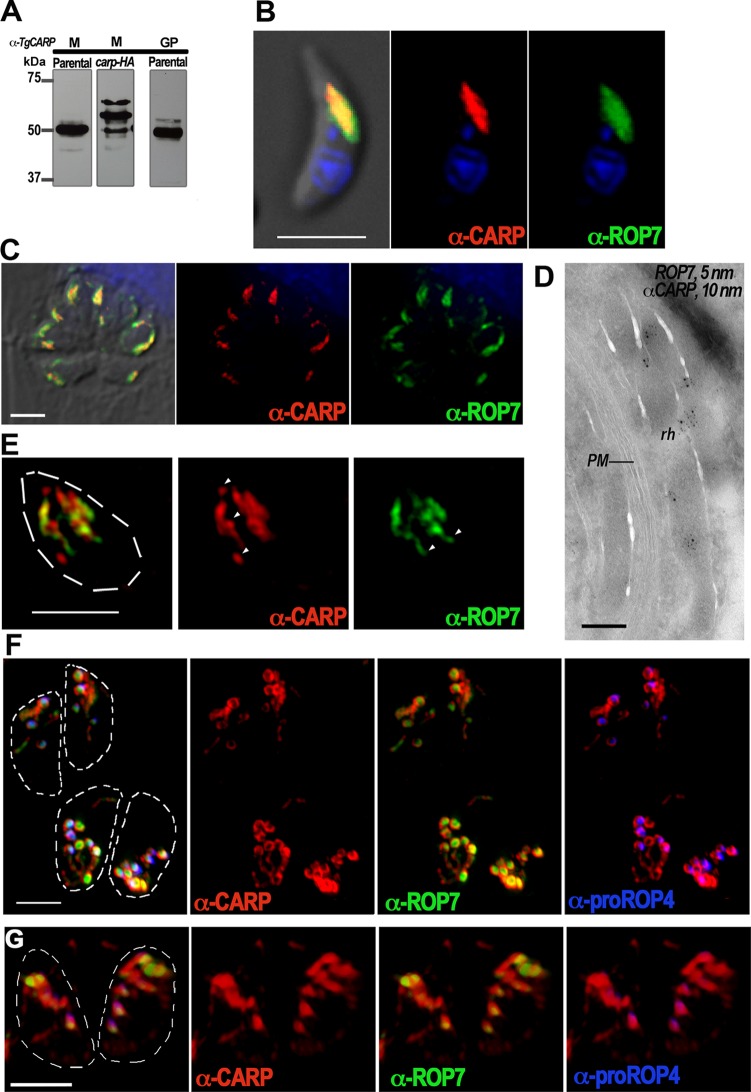
Localization of TgCA_RP with specific mouse and guinea pig antibodies. (A) Western blot analysis of lysates from RH *TATi Δku80* (parental) and *TgCA_RP-HA* (*carp-HA*) clonal lines using mouse (M) and guinea pig (GP) anti-TgCA_RP antibodies generated against the rTgCA_RP expressed in *E. coli*. A large band and two minor bands were observed in the parental lysate, while three distinct bands are observed in the *carp-HA* lysate. (B) IFA of an RH extracellular tachyzoite showing colocalization of anti-TgCA_RP with the rhoptry bulb marker anti-ROP7. (C) IFA of RH intracellular tachyzoites showing colocalization of anti-TgCA_RP with the rhoptry bulb marker anti-ROP7. (D) Cryo-immunoelectron microscopy of intracellular tachyzoites showing codistribution of TgCA_RP and ROP7 in the rhoptries (rh). PM, pellicle membrane. (E) SR-SIM images of an RH extracellular tachyzoite, showing partial colocalization in mature rhoptries, of TgCA_RP (red) and ROP7 (green). Certain regions (arrowheads) are distinct for TgCA_RP or ROP7 labeling. (F) IFAs of RH intracellular tachyzoites undergoing endodyogeny showing that anti-TgCA_RP (red) localizes specifically to the peripheral membranes of the nascent rhoptries, surrounding the labeling seen with anti-ROP7 (green) and the nascent rhoptry-specific antibody anti-proROP4 (blue). (G) In contrast, C-terminally tagged TgCA_RP-HA (red) localizes to the lumen of the nascent rhoptries, labeled with anti-ROP7 (green) and the nascent rhoptry-specific antibody anti-proROP4 (blue). Bars, 3 µm (B, C, and E to G) and 150 nm (D).

To investigate the localization of TgCA_RP in wild-type tachyzoites, we generated specific antibodies to recombinant TgCA_RP in mice (Fig. 3A, lanes M) and guinea pigs (Fig. 3A, lane GP). Western blot analysis of lysates from parental cell lines (*Δku80*) showed one prominent band flanked by faint upper and lower bands; the lower band was partially covered by the intense middle band (Fig. 3A, Parental). In contrast, Western blot analysis of lysates from the tagged cell line (*carp-HA*) showed three strong bands around the predicted size of 50 kDa, in agreement with the results obtained with anti-HA antibodies (Fig. 2B). The increased presence of these higher- and lower-molecular-mass forms suggested that the tag could be altering the maturation/processing of TgCA_RP (Fig. 3A, lanes M, Parental and *carp-HA*). Immunofluorescence assays (IFAs) using antibodies to TgCA_RP showed localization of the protein to the rhoptry bulbs of both extracellular (Fig. 3B) and intracellular (Fig. 3C) tachyzoites, as confirmed by its colocalization with ROP7 (Fig. 3B and C). This was further confirmed with cryo-immunoelectron microscopy (Fig. 3D). Using superresolution structured illumination microscopy (SR-SIM), we observed that TgCA_RP labels the bulbs of mature rhoptries. Interestingly, we observed regions of the rhoptry bulbs in which there was no colocalization between TgCA_RP and ROP7 (Fig. 3E, arrowheads). TgCA_RP localized specifically to the peripheral membrane of nascent rhoptries in dividing tachyzoites, surrounding the luminal proteins ROP7 and ROP4 (Fig. 3F). In contrast, TgCA_RP-HA did not specifically localize to the membrane of nascent rhoptries but instead showed labeling similar to ROP7 and ROP4 (Fig. 3G).

Tachyzoites pretreated with cytochalasin D successfully attach to host cells, but they are not able to invade. Under these conditions, rhoptry bulb contents are deposited in a trail of vesicles within the host cell cytosol. These vesicles were termed evacuoles because they do not contain parasites (31). We investigated if TgCA_RP was part of the evacuole contents according to a published protocol (31). Anti-TgCA_RP did not label evacuoles in attached RH strain tachyzoites, suggesting that TgCA_RP may not be secreted into the host cell (data not shown).

### TgCA_RP-knockout mutants exhibited reduced invasion *in vitro*.

**FIG 4:**
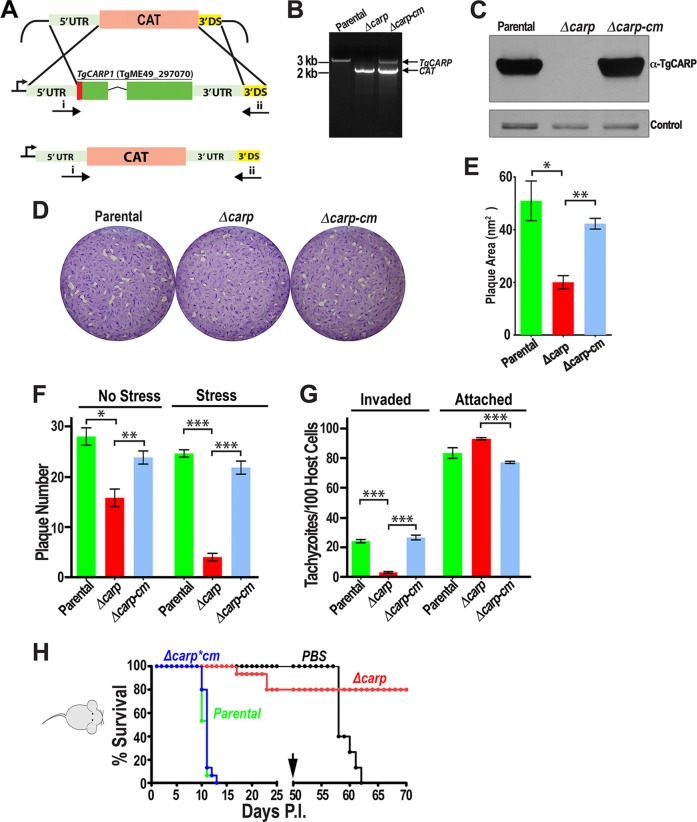
*Δcarp* mutants have a growth phenotype *in vitro* and *in vivo*. (A) Scheme showing the strategy used for creating *Δcarp* mutants. The endogenous gene was replaced with a chloramphenicol acetyltransferase (CAT) cassette via double homologous recombination. DS, downstream. (B) PCR amplification of genomic DNA extracted from parental, Δ*carp*, and Δ*carp-cm* clones after gene replacement showing the amplification of a 3.2-kb product corresponding to the *TgCA_RP* gene in the parental strain and a 2.1-kb product in the *Δcarp* mutant corresponding to the CAT cassette. Both bands are amplified in Δ*carp-cm*. Primers upstream (A, i) and downstream (A, ii) of the *CA_RP* gene were used for amplification. (C) Immunoblot assay of tachyzoite lysates with anti-TgCA_RP, showing the absence of TgCA_RP expression in the *Δcarp* mutant. Complementation of *Δcarp* mutants with a plasmid containing the *TgCA_RP* locus (*Δcarp-cm*), including the endogenous promoter, restored expression. (D) Representative plaque assay showing reduced plaque size formed by *Δcarp* mutant tachyzoites compared to parental (*Δku80*) and* Δcarp-cm* parasites (*n* = 3). (E) Quantification of parasite plaques showing smaller average area of *Δcarp* plaques. Student’s *t* test, *, *P* < 0.05; **, *P* < 0.005; ***; *P* < 0.0001 (means ± standard errors of the means, *n* = 3). (F) Plaquing efficiency assays showing that *Δcarp* parasites have a reduced invasion phenotype exacerbated by 1 h of extracellular stress in DMEM-HG medium. Student’s *t* test, *, *P* < 0.05; **, *P* < 0.005; ***, *P* < 0.0001 (means ± standard errors of the means, *n* = 3). (G) Red-green invasion assays showing reduced invasion by *Δcarp* tachyzoites. The “Invaded” bars show tachyzoites that attached and then successfully invaded, while the “Attached” bars show parasites that attached but failed to invade. Student’s *t* test, ***, *P* < 0.0001 (means ± standard errors of the means, *n* = 3). (H) Five outbred mice were inoculated intravenously with PBS or 50 parental (*Δku80*), *Δcarp*, or *Δcarp-cm* tachyzoites. Mouse cohorts inoculated with *Δcarp* mutants have increased survival compared to those inoculated with parental or *Δcarp-cm*. Surviving mice were protected against a challenge with 1,000 RH tachyzoites 50 days postinoculation (p.i.). The survival curve shows the combined results from 3 trials with 5 mice each (total of 15 mice per group).

To establish the role of TgCA_RP in the life cycle of *T. gondii*, we generated tachyzoite knockout mutants (*Δcarp*) by targeting the native *TgCA_RP* locus in the *Δku80* strain, which favors homologous recombination (32, 33) (Fig. 4A). Successful gene deletion was confirmed by PCR amplification of genomic DNA, extracted from subclones of the transfectants, using upstream and downstream primers (Fig. 4A; Table S3). A 2.1-kb product was amplified from the genomic DNA of a *Δcarp* mutant subclone, confirming the replacement of *TgCA_RP* with a chloramphenicol acetyltransferase (CAT) cassette (Fig. 4B). Successful gene deletion was confirmed by Western blot analysis of parasite lysates using mouse polyclonal antibody to TgCA_RP (Fig. 4C). Restoration of gene expression was achieved by transfecting *Δcarp* tachyzoites with a plasmid containing the entire *TgCA_RP* gene locus, including the predicted untranslated regions (UTRs) and the potential promoter region (500 bp upstream of the annotated 5′ UTR), which was amplified from genomic DNA via PCR. The expression of *TgCA_RP* in complemented mutants (*Δcarp-cm*) appeared to be at appropriate levels (Fig. 4C). *Δcarp-cm* mutants retained chloramphenicol resistance, and PCR of the genomic DNA showed bands of appropriate size for both the CAT cassette and the complemented *TgCA_RP* locus, confirming random integration (Fig. 4B). We compared plaque sizes of the parental (*Δku80*), knockout (*Δcarp*), and complemented (*Δcarp‑cm*) clones in parallel plaque assays and found a significant difference in plaque size (Fig. 4D and E). *Δcarp* parasites formed smaller plaques (Fig. 4D and E) and had an invasion defect, as shown by plaquing efficiency (Fig. 4F) as well as red-green invasion assays (Fig. 4G). The invasion defect observed in plaquing efficiency assays was exacerbated by extracellular stress consisting of a 1-h preincubation in Dulbecco’s modified Eagle’s medium (DMEM) (Fig. 4F). Interestingly, the number of *Δcarp* tachyzoites per vacuole was not affected compared to the number of tachyzoites formed by *Δku80* and *Δcarp‑cm* strains, after 25 h of intracellular growth (data not shown). These results indicated that invasion and not intracellular replication is the reason for reduced plaque size. This result is consistent with the fitness score of TgCA_RP, 0.31, which suggests that this gene is not relevant for intracellular replication (34).

10.1128/mSphere.00027-17.8 TABLE S3  Primers used in this work. Download TABLE S3, PDF file, 0.03 MB. Copyright © 2017 Chasen et al. 2017 Chasen et al. This content is distributed under the terms of the Creative Commons Attribution 4.0 International license.

### TgCA_RP is required for effective *in vivo* infection.

After establishing that the *Δcarp* mutant exhibited an *in vitro* growth phenotype, we tested the virulence of mutant tachyzoites in a mouse model. We first infected mice intraperitoneally (i.p.), but because of the highly virulent nature of the parental RH strain, it was difficult to see a clear difference in virulence when comparing survival between mutant and parental lines, although we did observe a small increase in survival time of mice inoculated with the *Δcarp* mutants (not shown). Dissemination through the circulation is important during natural infection, and this transit must expose tachyzoites to stressful conditions. Considering that the *Δcarp* mutant showed sensitivity to stress (Fig. 4F), we tested virulence by infecting mice intravenously (i.v.) (35). We inoculated mice i.v. with 50 tachyzoites of the *Δcarp*, parental, or *Δcarp-cm* strain. We observed that 70% of mice infected with *Δcarp* tachyzoites survived more than 30 days postinfection, while none of the mice infected with the parental or complemented parasites survived more than 12 days (Fig. 4H). Surviving mice and phosphate-buffered saline (PBS) control mice were challenged with 1,000 RH-rfp tachyzoites. All of the surviving mice previously inoculated with *Δcarp* tachyzoites survived the RH challenge without showing illness, in contrast to a 100% mortality of the PBS control mice (Fig. 4H). When mice were inoculated with 150 or 500 *Δcarp* tachyzoites, there was only a 40% and 10% survival rate, respectively, but there was also a delayed morbidity in mice that did not survive (Fig. S4). In conclusion, our results indicate that the infectivity of *Δcarp* tachyzoites is attenuated compared to the parental and *Δcarp-cm* strains.

10.1128/mSphere.00027-17.3 FIG S3  Cartoon showing locations of P5 to P10 used for the cloning of recombinant TgCA_RP. P5 and P6 amplified cDNA coding for the entire protein except for the signal peptide (red). P7 and P8 amplified the cDNA coding for the region from the Q in the putative SLL↓Q cleavage site (black line) to the GPI anchor omega site (orange line). P9 and P10 amplified only the region of cDNA coding for the carbonic anhydrase domain annotated by BLASTp. Download FIG S3, PDF file, 0.1 MB. Copyright © 2017 Chasen et al. 2017 Chasen et al. This content is distributed under the terms of the Creative Commons Attribution 4.0 International license.

10.1128/mSphere.00027-17.4 FIG S4  Five outbred mice were inoculated intravenously with 150 (top) or 500 (bottom) parental (*Δku80*), *Δcarp*, or *Δcarp-cm* tachyzoites. Mouse cohorts inoculated with *Δcarp* mutant parasites showed increased survival compared to those inoculated with parental or *Δcarp-cm* parasites. (Top) Data for 150 tachyzoites from a single experiment; (bottom) data from 500 tachyzoites, combined from two replicates. Download FIG S4, PDF file, 0.1 MB. Copyright © 2017 Chasen et al. 2017 Chasen et al. This content is distributed under the terms of the Creative Commons Attribution 4.0 International license.

### TgCA_RP is posttranslationally modified with a GPI anchor.

**FIG 5:**
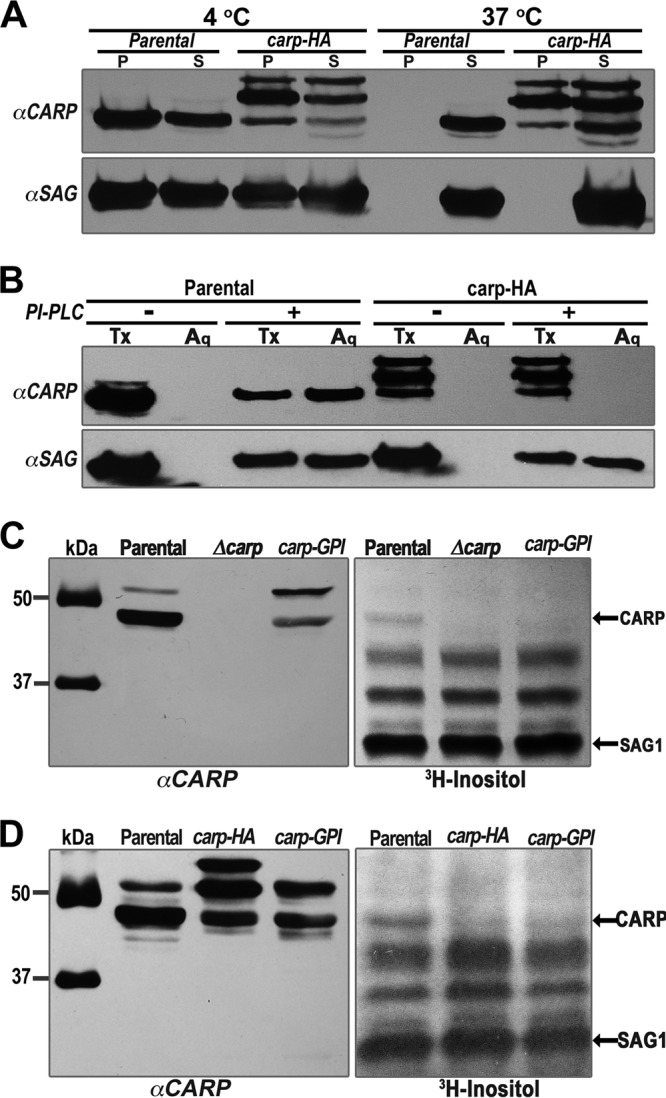
TgCA_RP has characteristics of a GPI-anchored protein and is labeled by myoinositol. (A) Tachyzoites were treated with 1% Triton X-100 at 4°C or 37°C. After centrifugation, immunoblot analysis of the pellet (P) and aqueous (S) fractions with anti-TgCA_RP revealed that TgCA_RP is only partially soluble in 1% Triton X-100 at 4°C and is completely soluble at 37°C. This behavior is mirrored by SAG1, a known GPI-anchored protein. The solubility of TgCA_RP-HA is largely unaffected by incubation at 37°C. (B) Immunoblot of tachyzoite lysates with anti-TgCA_RP after Triton X-114 detergent extraction. TgCA_RP is found in the detergent fraction (Tx) of mock-treated lysates (*−*) but is partially released into the aqueous fraction (Aq) when the lysate is treated with GPI-PLC (*+*) prior to extraction. SAG1, a known GPI-anchored protein, showed a similar pattern. C‑terminally tagged TgCA_RP-HA is not released into the aqueous fraction by GPI-PLC. (C) Autoradiography of lysates from parental and *Δcarp* tachyzoites labeled with [^3^H]myoinositol. The arrow points to a band of identical size as the one observed in the Western blot (anti-TgCA_RP) of the same membrane, and this band is absent in the lysate of *Δcarp* parasites. This absence supports the conclusion that TgCA_RP is GPI anchored. (D) The TgCA_RP band is also absent in autoradiography of *carp-HA* cells and mutants with a mutated GPI anchor attachment region, *ΔcarpGPI* (also in panel C).

To determine whether TgCA_RP is modified with a GPI anchor, we first investigated whether TgCA_RP was found in detergent-resistant membranes (DRMs). TgCA_RP was found in the pellet after incubation with 1% Triton X-100 at 4°C and in the soluble fraction after incubation at 37°C (Fig. 5A). This is consistent with the behavior of SAG1, a known GPI-anchored protein (36, 37) (Fig. 5A). TgCA_RP-HA showed no clear difference in solubility after 4°C or 37°C incubations, suggesting that it is not found in DRMs. We next investigated whether TgCA_RP is modified with a GPI anchor, by utilizing *Bacillus cereus* phosphatidylinositol-specific phospholipase C (PLC), an enzyme that specifically cleaves GPI anchors (38), followed by detergent extraction with Triton X-114. After PLC treatment and Triton X-114 phase separation, the aqueous and detergent phases were analyzed by Western blotting, probing for TgCA_RP or TgCA_RP-HA and for SAG1, as a GPI-anchored positive control (39). TgCA_RP and SAG1 were both detected in the aqueous phase after treatment with PLC but not after mock treatment, providing strong evidence for the GPI anchor modification of TgCA_RP. TgCA_RP-HA was detected only in the detergent phase, behaving as an integral membrane protein, but this behavior did not change after PLC treatment, suggesting that TgCA_RP-HA is not GPI modified, which could be a consequence of the disruption of the GPI consensus domain by the C-terminal tag (Fig. 5B). To radiolabel the GPI anchor of TgCA_RP, we grew mutants (*carp-HA* and *Δcarp*) and parental tachyzoites in the presence of [^3^H]inositol. Western blot analysis of lysates was performed with anti-TgCA_RP, and the membranes were subsequently exposed to X-ray film for autoradiography. [^3^H]inositol labeled several bands, which probably correspond to other GPI-modified proteins, such as the surface antigen SAG1 (39). A band was observed in lysates of the parental strain that corresponded to the band labeled by TgCA_RP on the Western blot. This band was absent in lysates from the *Δcarp* tachyzoites (Fig. 5C, right panel). Furthermore, this band was not observed in the autoradiography of lysates from [^3^H]inositol-labeled *carp-HA* mutants (Fig. 5D, right panel), supporting our hypothesis that the hemagglutinin (HA) tag interferes with the GPI modification. We also complemented *Δcarp* tachyzoites with a *TgCA_RP* that was modified by replacing the predicted GPI anchor cleavage site with a randomized amino acid sequence (*ΔcarpGPI*) (Fig. S4). [^3^H]inositol did not label TgCA_RP in these mutants (Fig. 5C and D). In summary, our results support the conclusion that TgCA_RP is GPI anchored. The results with *carp-HA* indicate that the C‑terminal 3×HA tag may disrupt the GPI anchor modification, despite its distance from the predicted GPI anchor attachment site. This phenomenon has been previously described for the *Plasmodium* protein PfASP (40).

### Rhoptry morphology is altered in TgCA_RP-knockout mutants.

**FIG 6:**
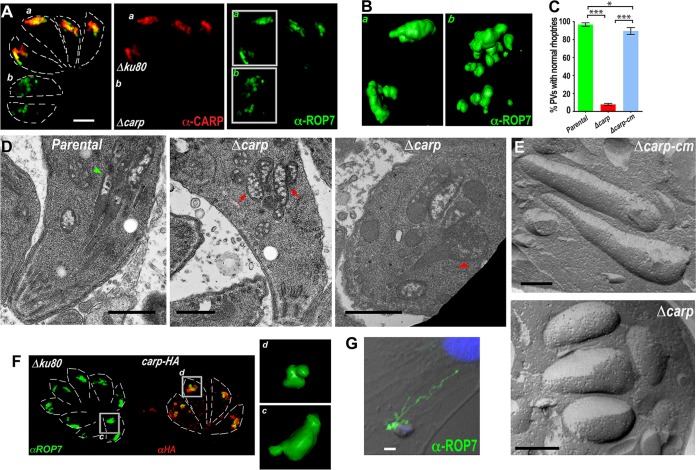
Rhoptry biogenesis is disrupted in* Δcarp* tachyzoites. (A) IFA of parental and *Δcarp* tachyzoites infecting the same host cell that were analyzed using SR-SIM followed by deconvolution and 3D reconstruction to reveal typical rhoptry bulb morphology in parental tachyzoites (a) and fragmentation of *Δcarp* tachyzoite rhoptry bulbs (b). (B) Enlargement of the highlighted areas shown in panel A (anti-ROP7). (C) Quantification revealed a significant difference between the percentages of vacuoles containing typical rhoptry morphology in host cell slides infected with parental tachyzoites and those infected with *Δcarp1* mutants. Complementation of the *TgCA_RP* gene locus (*carp-cm*) restored proper rhoptry morphology. More than 100 vacuoles were counted per assay. Student’s *t* test, *, *P* < 0.05; ***, *P* < 0.0001 (means ± standard errors of the means, *n* = 3). (D) Transmission electron microscopy of intracellular tachyzoites showed typical thin and elongated rhoptry bulb morphology in a parental tachyzoite (left, green arrowhead) and a representative example of the atypical fragmented rhoptry bulbs that do not connect to a rhoptry neck structure found in the majority of *Δcarp* tachyzoites (middle, red arrows). A minority of *Δcarp* tachyzoites contained fully formed rhoptry bulb and neck structures, but in these cases, the rhoptry bulb is atypically swollen (right, red arrowhead). (E) Freeze fracture electron microscopy of *Δcarp-cm* extracellular tachyzoites (top) shows typical elongated rhoptries in contrast to the short and thick rhoptries seen in the *Δcarp* mutants (bottom). (F) IFAs of parental (left) and *carp-HA* (right) tachyzoites infecting the same host cell labeled with anti-ROP7 (green) and anti-HA (red) that were analyzed using SR-SIM followed by deconvolution and 3D reconstruction to reveal typical rhoptry bulb morphology in parental tachyzoites (c) and atypical rhoptry morphology in *carp-HA* tachyzoites (d). (G) IFA of an evacuole secretion assay, which shows a *Δcarp* tachyzoite successfully secreting evacuoles labeled by anti-ROP7. This suggests that *Δcarp* rhoptries are capable of secreting rhoptry evacuoles after treatment with cytochalasin D.

Immunofluorescence analysis of *Δcarp* intracellular tachyzoites (Fig. 6A, b) using superresolution structured illumination microscopy revealed dramatic changes in rhoptry labeling compared to that of parental tachyzoites (Fig. 6A, a) infecting the same host cell monolayer. Three-dimensional (3D) reconstruction shows the fragmented nature of the rhoptries in the *Δcarp* tachyzoites (Fig. 6B). The percentage of parasitophorous vacuoles (PVs) containing at least one “normal” linear rhoptry (rhoptries in Fig. 3B) was significantly reduced in *Δcarp* tachyzoites compared to parental and *Δcarp-cm* parasites (Fig. 6C). PVs containing nascent rhoptries were labeled with the marker anti-proROP4 and excluded from counting. Transmission electron microscopy of intracellular tachyzoites revealed typical rhoptry morphology with a bulb and neck in parental tachyzoites, in contrast to the fragmented globular rhoptries, which do not connect to a rhoptry neck structure, in the majority of *Δcarp* tachyzoites (Fig. 6D). A minority of *Δcarp* tachyzoites did show a connected rhoptry bulb and neck, but the rhoptry bulb in these cases was swollen (Fig. 6D). Freeze fracture electron microscopy of *Δcarp* extracellular tachyzoites provided further evidence of the stunted rhoptry morphology compared to the elongated rhoptries of the *Δcarp-cm* mutants (Fig. 6E). Similar rhoptry morphology defects were observed in *carp-HA* tachyzoites (Fig. 6F).

To establish whether the rhoptries of *Δcarp* tachyzoites were still capable of secreting ROP proteins into the host cell, we performed an evacuole secretion assay with the *Δcarp* mutants. *Δcarp* tachyzoites were capable of secreting evacuoles (labeled with anti-ROP7) into the host cell, suggesting that the rhoptries of *Δcarp* tachyzoites are functional in secretion despite their altered morphology (Fig. 6G).

## DISCUSSION

We report here that the gene *TgCA_RP*, present in the *T. gondii* genome, encodes a catalytically inactive carbonic anhydrase-related protein (CARP) that is closely related to the recently identified η-class carbonic anhydrase present in *P. falciparum* (PfCA) (10, 11). Given that the closest active relative of TgCA_RP, PfCA, shares only 26% sequence identity, it was not surprising that our attempts to reconstitute activity by changing 1 to 2 amino acids of the Zn^2+^ binding domain with His were insufficient to restore activity to rTgCA_RP. PfCA is the only active member of the η-class that has been characterized, and no mutagenesis studies have been performed to identify essential residues. There are likely important residues for catalytic activity in η-class CAs other than the Zn^2+^ coordination site.

TgCA_RP localizes to both mature and nascent rhoptries. An antibody to the proregion of ROP4 was used for identification and colocalization to the nascent rhoptry vesicles (41). TgCA_RP appears to localize to the outer membrane of nascent rhoptries, surrounding the luminal localization of ROP7 and ROP4, suggesting that TgCA_RP may possess a localization signal that is distinct from other rhoptry bulb proteins. TgCA_RP is posttranslationally modified at its C-terminal region with a glycosylphosphatidylinositol (GPI) anchor, and blocking this C‑terminal region with an epitope tag altered localization in intracellular but not extracellular tachyzoites. Tachyzoites lacking TgCA_RP display a growth and invasion phenotype *in vitro*, have atypical rhoptry morphology, and exhibit reduced virulence in a mouse model. Ionic stress is a major challenge that *T. gondii* confronts when exiting the host cell into the extracellular medium. Dealing with these stressful changes is important for the survival of the parasite, which needs to actively invade other host cells to continue its lytic cycle. Tachyzoites lacking TgCA_RP showed increased sensitivity to extracellular stress, perhaps because poorly developed rhoptries are less stable and more difficult to maintain in a functional state for invasion.

While several GPI-anchored proteins that localize to secretory organelles in apicomplexan parasites have been described, TgCA_RP is the first GPI-anchored protein to localize to the rhoptry bulb of *T. gondii*. Rhoptry-associated membrane antigen (RAMA) and Pf34, both GPI-anchored proteins, localize to the bulb and neck of the *P. falciparum* rhoptries, respectively, while *T. gondii* subtilisin (TgSUB1) localizes to the micronemes (42). The atypical sushi protein (ASP) of *P. falciparum* (orthologous to *T. gondii* RON1) is also GPI anchored and localizes to the rhoptry neck (43). Other *T. gondii* RON proteins are predicted to have a GPI anchor as well, although this has not been experimentally verified (44). The propensity to associate with DRMs has been shown to be important for the appropriate trafficking of some GPI anchor proteins through the secretory pathway (45).

Parasites expressing the tagged version of TgCA_RP in *carp-HA* mutants express TgCA_RP without the GPI anchor. This conclusion is based on the results of two experiments. The first result was the failure of TgCA_RP-HA to be released into the aqueous phase by pretreatment of the parasite lysate with GPI-PLC, while both surface antigen SAG1 and TgCA_RP were released by this method. Second, [^3^H]inositol did not label TgCA_RP-HA in *carp-HA* tachyzoites. Disruption of the TgCA_RP GPI anchor attachment domain by the HA tag resulted in mislocalization of a large portion of TgCA_RP-HA protein in intracellular tachyzoites and a rhoptry biogenesis phenotype identical to the one obtained after deletion of the *TgCA_RP* gene. This defect was not rescued by the partial localization of TgCA_RP-HA to nascent rhoptries. The localization of TgCA_RP-HA was diffuse, and we did not see the clearly defined localization to the periphery of the nascent rhoptries as observed in the parental cell line. Our hypothesis is that the GPI anchor is acting as a localization signal. It is possible that the outer membrane of the nascent rhoptries has a different lipid composition, which favors the specific localization of GPI-anchored proteins (46). The presence of TgCA_RP specifically at the nascent rhoptry membrane or the GPI anchor itself may be necessary for its role in rhoptry biogenesis. We attempted to isolate *T. gondii* lipid rafts using a density gradient, but the results were not interpretable due to the lack of appropriate lipid raft markers for these parasites. However, we found that TgCA_RP separates with detergent-resistant membranes, typically enriched in lipid raft proteins (47–49).

There is evidence that CARPs coordinate the function of other proteins by protein-protein interaction (9). A BLASTp search of the TgCA_RP sequence against the human proteome showed that the CA-like domain of the gamma-type protein tyrosine phosphatase matches TgCA_RP with the highest similarity score. This protein acts as a hydrophobic binding pocket for contactin, a GPI-anchored neuronal surface protein that is important for cell adhesion and outgrowth (50, 51). A similar attachment role has been attributed to the CA-like N-terminal domain of the vaccinia virus (9). In addition, some CARPs interact with calcium channels (52). It is possible that TgCA_RP interacts in a similar way with an unknown binding partner in the nascent rhoptry membranes, promoting adhesion and fusion of the nascent rhoptry vesicles to form the mature organelle. The region containing the annotated CA domain of TgCA_RP was previously shown to be exposed to the cytosol, which would be consistent with this hypothesis (30). In this hypothetical scenario, the absence of TgCA_RP would cause a delay in the assembly of nascent rhoptry vesicles, which would become disorganized. This would explain the delayed maturation and unusual shape of the rhoptries that we observed in our extracellular *Δcarp* mutants.

In summary, this is the first report of a GPI-anchored CARP in *T. gondii*. TgCA_RP localizes to the outer membrane of nascent rhoptries, where it plays a role in the biogenesis of the organelle. TgCA_RP appears to be important for rhoptry shape and maturation. When TgCA_RP is not expressed, parasites become less fit for growth *in vitro* and *in vivo*. Rhoptries in these mutants are probably suboptimally functional. An interesting and unique aspect of TgCA_RP is its similarity to the η-class carbonic anhydrase described in *Plasmodium*, and its inactivity, even when the proposed Zn^2+^ coordinating histidine residues were restored. Our interpretation is that other features, apart from the proposed Zn^2+^ coordination domain, differentiate TgCA_RP from PfCA. The *T. gondii* CARP is the first CARP described in protists that does not belong to the α-class, suggesting that the evolutionary origin of these carbonic anhydrase-related proteins may be earlier than previously anticipated.

## MATERIALS AND METHODS

### Analysis of sequence and alignment.

Alignment was performed using the T-Coffee web server (53). One unified alignment was performed, and the sequences used are shown in Table S1 in the supplemental material. The resulting alignment was edited in Jalview2 (54). Poorly aligned N‑terminal regions were trimmed.

### Parasite cultures and generation of mutants.

Tachyzoites of the *T. gondii* RH *Δku80 Δhxgprt* (33) and RH *Δku80 TATi* (55) strains were cultured in human foreskin fibroblasts (HFF) or hTert (human telomerase reverse transcriptase) fibroblasts in Dulbecco’s modified Eagle’s medium (DMEM) supplemented with 1% fetal bovine serum (FBS), 1 mM sodium pyruvate, and 2 mM glutamine. Tachyzoites were obtained from infected hTert cells by passing them through a 25-gauge needle or otherwise collected from the supernatant of infected cells after natural egress. The RH *Δku80 TATi* strain was obtained from Boris Striepen (University of Georgia), and the RH *Δku80 Δhxgprt* strain was obtained from David Bzik (Dartmouth Medical School).

For C-terminal tagging of the *TgCA_RP* gene, the 3′ 1,459 bp (minus the stop codon) of the gene annotated as a hypothetical protein, TgME49_297070, was amplified using primers P1 and P2 (see Table S3 in the supplemental material), which added the sequence required for ligation-independent cloning. The PCR product was purified using the Qiaex II gel extraction kit (Qiagen) and cloned into the pLIC-3×HA-CAT plasmid. The purified PCR product and plasmid were treated and combined as described in the work of Huynh and Carruthers (33). Eighty micrograms of the sterilized plasmid pTgCA_RP-3×HA-CAT was transfected into 1 × 10^7^ RH *Δku80 TATi* (56). Transfected tachyzoites were selected with 20 µM chloramphenicol, and clones were isolated by limiting dilution. Genomic DNA of clones was isolated and screened by PCR using a primer upstream of the original amplification from *TgCA_RP* using forward primer P3 and a downstream pLIC-3×HA-CAT reverse primer, P4. Clones were further confirmed by Western blot analysis.

Deletion of the *TgCA_RP* gene was achieved by transfecting *Δku80 Δhxgprt* tachyzoites with a plasmid (pCTY) containing a chloramphenicol cassette flanked by a 1-kb region of the *TgCA_RP* 5′ UTR and 1 kb downstream of the 3′ UTR. The parasites were selected with chloramphenicol followed by subcloning. Complementation of *TgCA_RP* was accomplished by cloning the *TgCA_RP* gene, including the UTRs and potential promoter region, into the pDTM3 plasmid. The construct was transfected into *Δcarp* tachyzoites and selected using pyrimethamine, followed by subcloning.

### *TgCA_RP* expression.

*TgCA_RP* was expressed in *E. coli* without the 36-amino-acid signal peptide. A PCR product was generated by amplifying a DNA fragment from *T. gondii* cDNA with primers P5 and P6 (Table S3). The fragment was cloned into the pTRCHisB expression vector (Invitrogen) using BamHI and XhoI restriction sites. The TgCA_RP-pTRCHis construct was sequenced and transformed into *E. coli* BL21(DE3) RIPL. Cells were induced for 2 h at 37°C with 1 mM isopropyl-β-d-thiogalactopyranoside (IPTG). The inclusion bodies containing rTgCA_RP were isolated using the protocol described in the GE Healthcare recombinant protein purification manual. Inclusion bodies were solubilized with 0.02% SDS in PBS, pH 11. pH was adjusted to 7.4 prior to the inoculation.

For enzyme activity, soluble recombinant protein (TgCA_RP) was expressed using the Pet32Lic/EK vector (Novagen) system. Two fragments from *TgCA_RP* of the previously amplified TgCA_RP cDNA were used. rTgCA_RPa (aa 121 to 445) was amplified using P7 and P8, and rTgCA_RPb (aa 94 to 488) was amplified using primers P9 and P10 (Table S3; Fig. S2). Mutagenesis of F233H (primers P11 and P12) and Q254H (primers P13 and P14) (Table S3) was performed using the QuikChange mutagenesis kit (NEB) and confirmed via sequencing. LB was supplemented with 125 μM ZnSO_4_ prior to induction with IPTG to ensure adequate Zn^2+^ for proper folding of the recombinant CA.

### Carbonic anhydrase activity assays.

The *p*-nitrophenyl acetate hydrolysis assays were performed as described by Armstrong et al. (26) with some modifications. A fresh 3 mM stock pNPA solution was prepared for each experiment by dissolving the compound in acetone followed by water dilution to the final concentration of 3 mM. Readings were taken in a plate or cuvette in a SpectraMax e^2^ plate reader at the isosbestic wavelength (348 nm) of *p*-nitrophenol and the *p*-nitrophenylate ion. Numerous buffers (25 mM Tris-HCl, 25 mM Tris-SO_4_, 25 mM Tricine-KOH, 25 mM HEPES-KOH, 50 mM MES, and 50 mM MOPS) and a range of pH conditions (6.0 to 8.0) were tested. In-gel CO_2_ hydration assays were performed as described by Ramanan et al. (27) with the addition of 1 μM ZnSO_4_ in the Triton X-100 refolding buffer. Commercially available bovine carbonic anhydrase (Sigma catalog no. C3934) was used as a positive control for activity.

### Antibody production and affinity purification.

Antibodies to recombinant TgCA_RP were generated in mice and guinea pigs. Mouse antibodies were produced via intraperitoneal inoculation of six CD-1 mice (Charles River, Inc.) with 100 μg of rTgCA_RP. Mice were boosted twice with 50 μg of rTgCA_RP. Final serum was collected by cardiac puncture after CO_2_ euthanasia. Guinea pig antibodies were produced via subdermal inoculation of Hartley strain guinea pigs (Charles River, Inc.) with 200 μg of solubilized rTgCA_RP and mixed with complete Freund’s adjuvant. Guinea pigs were boosted twice with 100 μg of solubilized rTgCA_RP. Final serum was collected using cardiac puncture. Inoculations and final serum collections were performed under anesthesia with isoflurane.

The antibodies used in IFAs were either affinity purified (mouse anti-TgCA_RP) or affinity purified and adsorbed (guinea pig anti-TgCA_RP). Adsorption was performed by cross-linking lysate from *Δcarp* tachyzoites to cyanogen bromide-activated resin (Sigma) according to the commercial Sigma protocol. One milliliter of anti-TgCA_RP guinea pig serum was incubated with beads for 2 h at room temperature, and the supernatant was collected for affinity purification. Affinity purification was performed by running 500 µg of purified inclusion bodies on a single-well SDS-PAGE gel followed by transfer to a nitrocellulose membrane. The TgCA_RP band region was excised and incubated overnight with 200 µl (mouse) or 1 ml (guinea pig) of serum diluted with 5 ml of incubation buffer (50 mM Tris-HCl, pH 10.2, 0.45 M NaCl, 0.01% Tween 20). The membrane was washed with PBS, pH 7.4. Antibody was eluted from the membrane twice using 500 µl of stripping buffer (0.2 M glycine-HCl, pH 2.4, 0.5 M NaCl), which was pipetted over the membrane for 2 min and then equilibrated to pH 7.5 with 1 M Tris-HCl, pH 9.5.

### Western blot analysis and immunofluorescence assays.

Purified tachyzoites were treated with cell lysis buffer M (Sigma) and 25 units of Benzonase (Novagen) for 5 min at room temperature followed by addition of an equal volume of 2% SDS-1 mM EDTA solution. Total protein was quantified with a NanoDrop spectrophotometer (Thermo Scientific). Samples were resolved using a 10% *bis*-acrylamide gel in a Tris-HCl SDS buffer system (Bio-Rad). Gels were either transferred for Western blot analysis or stained with amido black. Primary antibody dilutions were as follows: anti-HA, 1:50 (monoclonal rat; Roche); mouse anti-TgCA_RP, 1:1,000; guinea pig anti-TgCA_RP, 1:500. Secondary horseradish peroxidase (HRP) antibodies were used at 1:10,000 dilutions.

Indirect immunofluorescence assays (IFAs) were performed on either naturally egressed tachyzoites or infected human foreskin fibroblast (HFF) monolayers. Parasites or monolayers were washed once using buffer A with glucose (BAG; 116 mM NaCl, 5.4 mM KCl, 0.8 mM MgSO_4_, 50 mM HEPES, pH 7.2, and 5.5 mM glucose) and then fixed with 3% formaldehyde for 15 min, followed by permeabilization using 0.25% Triton X-100 for 10 min and blocking with 3% bovine serum albumin. Labeling was performed as previously described (57). Images were collected using an Olympus IX-71 inverted fluorescence microscope with a photometric CoolSNAP HQ charge-coupled device (CCD) camera driven by DeltaVision software (Applied Precision, Seattle, WA). Superresolution images were collected using the Elyra S1 superresolution structured illumination microscopy system (Zeiss), and 3D reconstructions were derived using Volocity (Perkin-Elmer). Dilutions used were as follows: anti-ROP7 mouse, 1:2,000; anti-proROP4 (UVT-70, 1:500), 1:2,000; anti-TgCA_RP guinea pig, 1:50; anti-TgCA_RP mouse, 1:50; and anti-HA rat (Roche), 1:50. Anti-ROP7 and anti-proROP4 were provided generously by Peter Bradley and Gary Ward, respectively.

### Immunoelectron microscopy.

Extracellular *T. gondii* endogenously expressing the C-terminal 3×HA tag (TgCA_RP-HA) was washed twice with PBS before fixation in 4% paraformaldehyde (Electron Microscopy Sciences, PA) in 0.25 M HEPES (pH 7.4) for 1 h at room temperature and then in 8% paraformaldehyde in the same buffer overnight at 4°C. Parasites were pelleted in 10% fish skin gelatin, and the gelatin-embedded pellets were infiltrated overnight with 2.3 M sucrose at 4°C and frozen in liquid nitrogen. Ultrathin cryosections were incubated in PBS and 1% fish skin gelatin containing mouse anti-HA antibody at a 1/5 dilution and then exposed to the secondary antibody that was revealed with 10-nm protein α-gold conjugates. Sections were observed and images were recorded with a Philips CM120 electron microscope (Eindhoven, the Netherlands) under 80 kV. For confirmation of rhoptry localization of TgCA_RP, ultrathin cryosections of *T. gondii-*infected cells were processed as described for extracellular parasites and immunolabeled with guinea pig anti-TgCA_RP and mouse anti-ROP7 antibodies at 1:25 and 1:750 dilutions, respectively, and detected with protein A-gold conjugates of 10 nm for TgCA_RP and 5 nm for ROP7.

### Invasion and growth assays.

Red-green invasion assays were performed as described in reference 58 with modifications. The number of tachyzoites used was 2 × 10^7^, and invasion was for 5 min. Plaque growth and plaquing efficiency assays were performed as previously described (35) with modifications. For plaque growth assays, 125 tachyzoites were used for infection of hTert fibroblasts followed by an incubation time of 10 days prior to fixing and staining. Plaquing efficiency assay plates were infected with 2,000 tachyzoites for 1 h prior to washing and then allowed to incubate for 6 days prior to fixation and staining with crystal violet. For intracellular growth assays, coverslips with hTert fibroblasts were cooled to 4°C and infected with 5 × 10^5^ tachyzoites. Coverslips were first incubated for 15 min on ice followed for 20 min at 37°C. Coverslips were washed three times with sterile PBS. Fresh DMEM, high glucose (DMEM HG), plus 1% fetal bovine serum (FBS) was added and incubated at 37°C for 25 h prior to fixation with 3% paraformaldehyde. Giemsa staining was done as described previously (61). Vacuoles containing 2, 4, 8, 16, and 32 tachyzoites were counted.

### Virulence tests in mice.

For each experiment, cohorts of 5 female adult Swiss Webster mice (Charles River, Inc.) were inoculated intravenously via the tail vein with PBS or 50, 150, or 500 tachyzoites of the parental (*Δku80* Δ*hxgprt*), *Δcarp*, or *Δcarp-cm* strain. The tachyzoites were mechanically released via syringe from the host cell monolayer and resuspended in 100 μl of sterile PBS at the proper concentration. Mice were observed several times daily. Mice were humanely euthanized when the illness score exceeded the level deemed acceptable by the established animal use protocol. Mice that survived the initial challenge with 50 tachyzoites were rechallenged by intraperitoneal inoculation with 1,000 RH-rfp tachyzoites, examined daily for signs of illness, and euthanized if serious illness was observed. Mouse experiments in this work followed a reviewed and approved protocol by the Institutional Animal Care and Use Committee (IACUC). Animal protocols followed the U.S. Government principles for the Utilization and Care of Vertebrate Animals. The protocol was reviewed and approved by the University of Georgia IACUC (protocol number A2015-02-025-R2).

### PI-PLC treatments and detergent extractions.

Naturally egressed tachyzoites were lysed via freeze-thawing and treated (or mock treated) with 0.4 units of GPI-specific phospholipase C (PI-PLC) from *Bacillus cereus* (Sigma) for 2 h at 37°C. Detergent extractions and acetone precipitations were performed as previously described (42). Isolation of detergent-resistant domains from naturally egressed tachyzoites was performed as previously described (59).

### *myo*-[^3^H]inositol labeling.

Parental (*Δku80 Δhxgprt*), *Δcarp*, and *carp-HA* tachyzoites were used to infect hTert monolayers in T12.5 flasks containing DMEM–high-glucose medium with 1% FBS. The monolayer was washed 24 h after the initial infection with Hanks balanced salt solution, and the medium was replaced with DMEM HG without inositol (MP Biologicals) containing 100 μCi *myo*-(2,3)-[^3^H]inositol (American Radiolabeled Chemicals). Parasites were allowed to egress naturally (~18 h) and were purified and washed as described previously (57). SDS-PAGE and Western blot analyses followed, and the nitrocellulose membranes were exposed to a radiography film (Denville Scientific) for 40 days before development.

### Rhoptry morphology.

For freeze fracture analysis, tachyzoites were fixed in 2.5% glutaraldehyde and processed as previously described (60). The material was washed in 0.1 M sodium cacodylate buffer and infiltrated in ascending concentrations of glycerol. The cells were maintained in 15% (vol/vol) glycerol for 12 h at 4°C. Cells were then infiltrated with 30% glycerol for 3 h, pelleted, mounted on support disks, and frozen in the liquid phase of Freon 22 cooled by liquid nitrogen. Frozen specimens were transferred and fractured in a BAF 060 freeze fracture apparatus (Baltec). Immediately after fracturing, the exposed surfaces were shadowed with platinum at an angle of 45° and carbon at an angle of 90°. Metal replicas were cleaned in sodium hypochlorite, collected on Formvar-covered nickel grids, and examined in a Tecnai Spirit Biotwin microscope (FEI, the Netherlands) operating at 120 kV.

For routine transmission electron microscopy, cells were fixed in 2.5% glutaraldehyde and postfixed in 1% osmium tetroxide, 1.25% potassium ferrocyanide, and 5 mM CaCl_2_ in 0.1 M cacodylate buffer (pH 7.2). Samples were washed in the same buffer, dehydrated in an ascending acetone series, and embedded in epoxy resin. Ultrathin sections were collected and observed in a JEOL JEM-1210 transmission electron microscope (JEOL, Tokyo, Japan).

For rhoptry morphology quantification assays, tachyzoites were allowed to invade host cell monolayers on glass coverslips. After 24 h, infected cells were fixed and IFAs was performed with anti-ROP7 as described above. For each coverslip, 100 parasitophorous vacuoles were counted. A vacuole containing at least one rhoptry of “normal,” linear morphology (Fig. 3B) was counted as normal.

### Rhoptry evacuoles.

Evacuoles were examined as previously described (31) with some modifications. Approximately 4 × 10^6^ naturally egressed *Δcarp* tachyzoites were resuspended in 1.5 ml invasion medium (DMEM HG, 3% FBS, 10 mM HEPES, pH 7.4) with 1 µM cytochalasin D (Sigma-Aldrich, St. Louis, MO, USA) and incubated at room temperature for 10 min. After incubation, 750 μl was transferred to hTert fibroblast-coated coverslips and incubated at 37°C for 15 min. Following a PBS wash, the coverslips were fixed for 30 min at room temperature with 2.5% paraformaldehyde. Following fixation, parasites were incubated with permeabilization/blocking buffer (0.05% saponin, 10% FBS, PBS, pH 7.4) for 15 min at 37°C. IFAs were performed as described above.

### Statistical analyses.

All statistical analyses were performed using GraphPad Prism software (version 7).

10.1128/mSphere.00027-17.5 FIG S5  Region of the TgCARP amino acid sequence containing the predicted GPI anchor omega site (S490, blue) and surrounding region (GWFSAAGE). This GPI anchor attachment region was mutated to a randomly generated amino acid sequence (KIDEVARL, red) for complementation of *Δcarp* mutants to generate mutant TgCARP that is not GPI anchored. Download FIG S5, PDF file, 0.1 MB. Copyright © 2017 Chasen et al. 2017 Chasen et al. This content is distributed under the terms of the Creative Commons Attribution 4.0 International license.
