# Supplementary material for: A Glycosylphosphatidylinositol-Anchored Carbonic Anhydrase-Related Protein of Toxoplasma gondii Is Important for Rhoptry Biogenesis and Virulence
Source: mSphere. 2017 May 17;2(3):e00027-17. doi: 10.1128/mSphere.00027-17 (PMC5437132; doi:10.1128/mSphere.00027-17)
Supplement: FIG S1 [file sph003172284sf4.pdf]

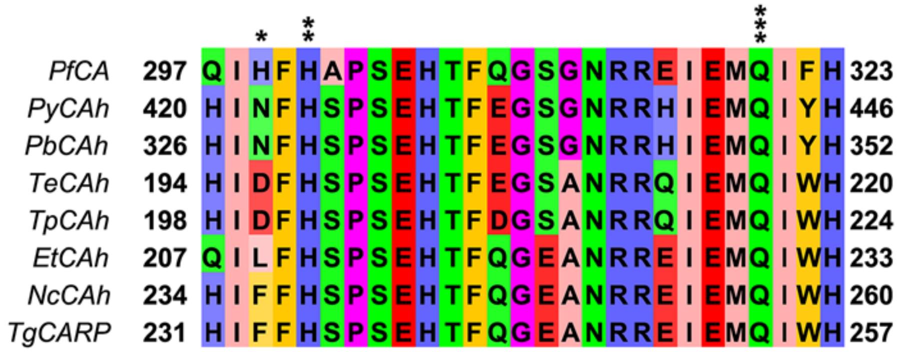
PfCA 297 QIHFHAPSEHTFQGSGNRREIEMQIFH 323
PyCAh 420 HINFHSPSEHTFEGSGNRRHIEMQIYH 446
PbCAh 326 HINFHSPSEHTFEGSGNRRHIEMQIYH 352
TeCAh 194 HIDFHSPSEHTFEGSANRRQIEMQIWH 220
TpCAh 198 HIDFHSPSEHTFDGSANRRQIEMQIWH 224
EtCAh 207 QILFHSPSEHTFQGEANRREIEMQIWH 233
NcCAh 234 HIFFHSPSEHTFQGEANRREIEMQIWH 260
TgCARP 231 HIFFHSPSEHTFQGEANRREIEMQIWH 257

FIG S1
