# Supplementary figures and images for: A Glycosylphosphatidylinositol-Anchored Carbonic Anhydrase-Related Protein of Toxoplasma gondii Is Important for Rhoptry Biogenesis and Virulence
Source: mSphere. 2017 May 17;2(3):e00027-17. doi: 10.1128/mSphere.00027-17 (PMC5437132; doi:10.1128/mSphere.00027-17)

Figure S2

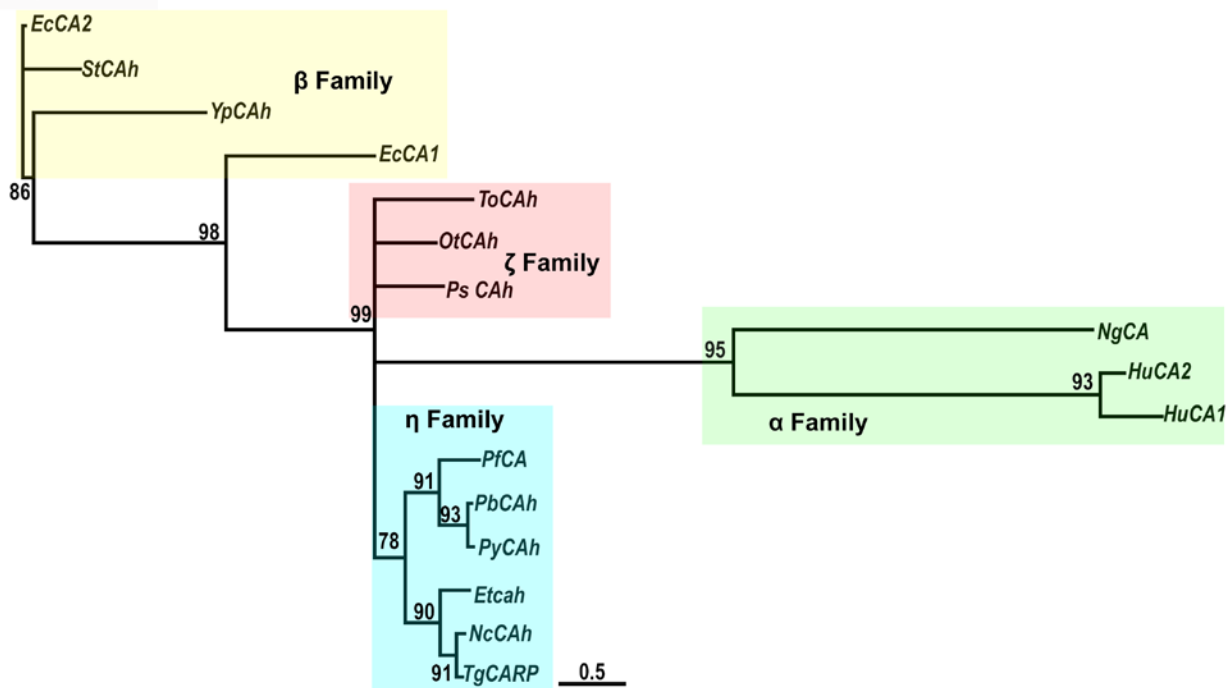

Supplement: FIG S2 [file sph003172284sf5.pdf]

Figure S3

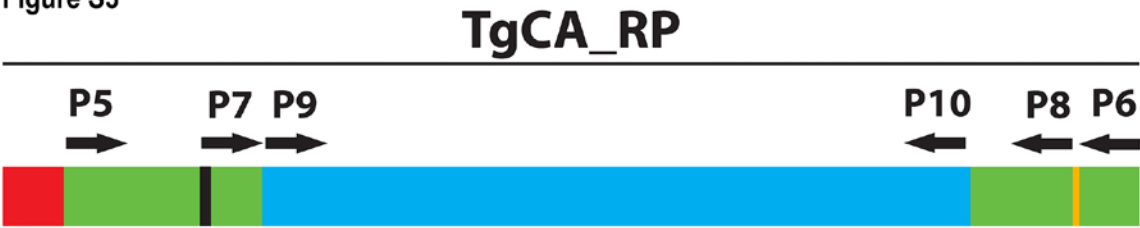

Supplement: FIG S3 [file sph003172284sf6.pdf]

Figure S4

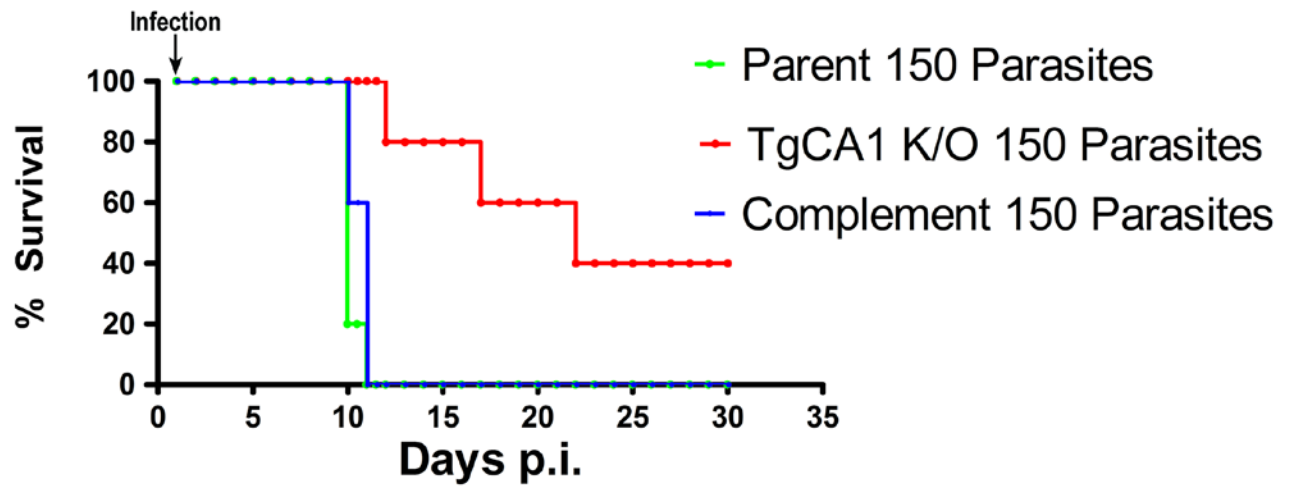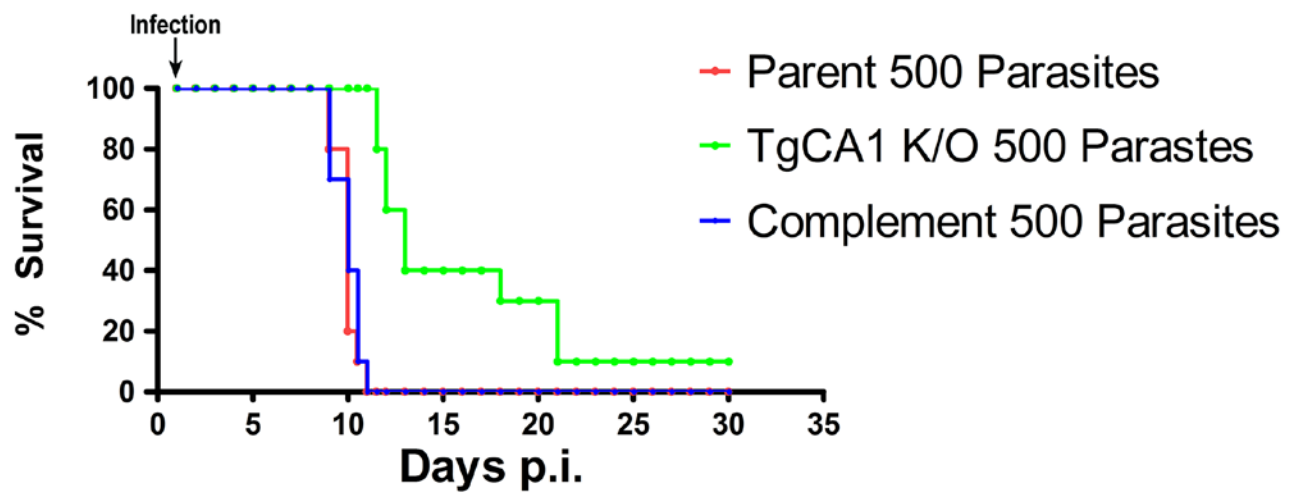

Supplement: FIG S4 [file sph003172284sf7.pdf]
