# Supplementary material for: A Glycosylphosphatidylinositol-Anchored Carbonic Anhydrase-Related Protein of Toxoplasma gondii Is Important for Rhoptry Biogenesis and Virulence
Source: mSphere. 2017 May 17;2(3):e00027-17. doi: 10.1128/mSphere.00027-17 (PMC5437132; doi:10.1128/mSphere.00027-17)
Supplement: TABLE S1 [file sph003172284st1.pdf]

Table S1

Organism	Accession number/ NCBI	EuPathDB ID	Reference
Homo sapiens, (Isoform II), *HuCA2*	AAH11949.1	--	1, 2
Homo sapiens, (Isoform I), *HuCA1*	NP_001158302.1	--	2
Neisseria gonorrhoeae, *NgCAh*	WP_003688976.1	NCLIV_006180	3
Plasmodium falciparum 3D7, *PfCA*	XP_001348081.2	PF3D7_1140000	4
Plasmodium yoelli 17XNL, *PvCAh*	XP_726574.1	PY00744	
Plasmodium berghei ANKA, *PbCAh*	XP_676575.1	PBANKA_090900	
Theileria equii, TeCAh	XP_004828973.1	BEWA_021550	
Theileria parva,TpCAh	XP_764978.1	TP02_0412	
Neospora caninum Liverpool, *NcCAh*	XP_003880177.1	NCLIV_006180	
Eimeria tenella Houghton, *EtCAh*	XP_013233689.1	ETH_00027955	
Toxoplasma gondii GT1, *TgCA_RP*	XP_002371137.1	TGME49_297070	
Thalassiosira oceanica, ToCdCAh	K0SZQ0		
Otreococcus tauri, OtCdCAh	A0A090N4Y7		
Psuedoalteromonas sp., PsCdCAh	A0A0A7EH23		
Escherichia coli,yadF, EcCA1	C3TN07		
Escherichia coli, EcCA2	P61517		
Salmonella typhimurium , StCAh	Q8ZRS0		
Yersinia pestis, yadF, YpCAh	Q0WBN9		

References:

1. Kiefer, L. L. & Fierke, C. A. Functional characterization of human carbonic anhydrase II variants with altered zinc binding sites. *Biochem.* **33**, 15233-15240 (1994).

2. Khalifah, R. G. The carbon dioxide hydration activity of carbonic anhydrase. I. Stop-flow kinetic studies on the native human isoenzymes B and C. *J. Biol. Chem.* **246**, 2561-2573 (1971).

3. Elleby, B., Chirica, L. C., Tu, C., Zeppezauer, M. & Lindskog, S. Characterization of carbonic anhydrase from Neisseria gonorrhoeae. *Eur. J. Biol. Chem./ FEBS* **268**, 1613-1619 (2001).

4. De Simone, G., Di Fiore, A., Capasso, C. & Supuran, C. T. The zinc coordination pattern in the eta-carbonic anhydrase from *Plasmodium falciparum* is different from all other carbonic anhydrase genetic families. *Bioorg .Med. Chem. Lett.* **25**, 1385-1389, (2015)
