# Supplementary material for: A Glycosylphosphatidylinositol-Anchored Carbonic Anhydrase-Related Protein of Toxoplasma gondii Is Important for Rhoptry Biogenesis and Virulence
Source: mSphere. 2017 May 17;2(3):e00027-17. doi: 10.1128/mSphere.00027-17 (PMC5437132; doi:10.1128/mSphere.00027-17)
Supplement: TABLE S2 [file sph003172284st2.pdf]

Table S2

Organism	EUPath ID	Pred-GPI	PredGPI Specificity	GPI-SOM
Toxoplasma gondii	TGME49_297070	Yes	99.5%	Yes
Neospora caninum	NCLIV_006180	Yes	99.5%	Yes
Eimeria tenella	ETH_00027955	Yes	100%	Yes
Theileria equii	BEWA_021550	Yes	99.8%	Yes
Theileria parva	TP02_0412	Yes	99.9%	Yes
Plasmodium falciparum	PF3D7_1140000	No	74.8%	No
Plasmodium yoelli	PY00744	Yes	99.6%	Yes
Plasmodium berghei	PBANKA_021550	Yes	99.1%	Yes
