# Supplementary material for: A Glycosylphosphatidylinositol-Anchored Carbonic Anhydrase-Related Protein of Toxoplasma gondii Is Important for Rhoptry Biogenesis and Virulence
Source: mSphere. 2017 May 17;2(3):e00027-17. doi: 10.1128/mSphere.00027-17 (PMC5437132; doi:10.1128/mSphere.00027-17)
Supplement: TABLE S3 [file sph003172284st3.pdf]

Table S3

Nº	Primer
1	5´_TACTTCCAATCCAATTTAATGCTACAGGCATGCAGCAGTCGCCAGTCG_3´
2	5´_TCCTCCACTTCCAATTTTAGCTATGCCAACAGTATTCAAGAAAACGAGGGC_3´
3	5´_GGCGGTTGGCTCCATCCCTCA_3´
4	5´_CGATACCGTCGACCTCGAGTAG_3´
5	5´_ggatccgACGCTAGCTTTTCATCGA_3´
6	5´_ctcgagTATGCCAACAGTATTCAAGAAAACGAGGGC_3´
7	5´_gacgacgacaagatgCAGGATGTGGCTGAGGAG_3´
8	5´_gaggagaagcccggttaCCAGCCTTGGAACATTTG_3´
9	5´_gacgacgacaagatgTGGGACTACAAGCAACATGG_3´
10	5´_gaggagaagcccggttaGTCTTCCACATTCTGCAGC_3´
11	5´_ACTGCTCAACACATCcatTTTCACTCCCCCTCTG_3´
12	5´_GTACATCTCCGTCGTGTCTGTGCTAAATAGTGC_3´
13	5´_AATTGAAATGcatATTTGGCACTATTCGAACG_3´
14	5´_TCACGCCGATTCGCCTCG_3´
