# Supplementary material for: A Glycosylphosphatidylinositol-Anchored Carbonic Anhydrase-Related Protein of Toxoplasma gondii Is Important for Rhoptry Biogenesis and Virulence
Source: mSphere. 2017 May 17;2(3):e00027-17. doi: 10.1128/mSphere.00027-17 (PMC5437132; doi:10.1128/mSphere.00027-17)
Supplement: FIG S5 [file sph003172284sf8.pdf]

Figure S5

WT 485SKEGAQMFQGWFSAAGEHTFGHSGTALAFATAALVFLNTVGI*
GPI1 485SKEGAQMFQKIDEVARLHTFGHSGTALAFATAALVFLNTVGI*
